# Biosynthesis, characterization of PLGA coated folate-mediated multiple drug loaded copper oxide (CuO) nanoparticles and it’s cytotoxicity on nasopharyngeal cancer cell lines

**DOI:** 10.1186/s13568-020-01096-2

**Published:** 2020-09-03

**Authors:** Long-Mei Guo, Xue-Mei Xu, Dong Zhao, Xun-Gong Cai, Bin Zhou

**Affiliations:** Department of Otolaryngology, The Fourth Affiliated Hospital of Harbin Medical University, No. 37 of Yiyuan Street, Nangang District, Harbin, 150001 Heilongjiang China

**Keywords:** Copper oxide, Nasopharyngeal, Cytotoxicity, Impediametric, Folate

## Abstract

Cytotoxicity of CuO nanoparticles (NPs) are an impediment in utilizing them as an effective nanocarriers of chemotherapeutic drugs for targeted drug delivery in nasopharyngeal cancer. In our current study, we have designed a two-step synthesis and coating of CuO NPs with different concentrations of PLGA (polylactide-co-glycolide) to reduce the cytotoxicity. This was further conjugated with folic acid to enhance targeting to specific tissue. The multiple drugs loaded in the NPs were two potent anticancer drugs doxorubicin and docetaxel. A complete characterization studies including micrographic analysis, zeta potential measurements, polydispersity index, Fourier transform infrared spectroscopy (FTIR), encapsulation and loading efficiencies, stability and in vitro release studies were done. Cytoxicity studies were done with MTT 3-(4,5-dimethylthiazol-2-yl)-2,5-diphenyltetrazolium bromide assay, acridine orange/ethidium bromide and DAPI (4, 6-diamidino-2-phenylindole, dihydrochloride) staining procedures. Impediametric studies were also carried out to reinforce the reduction in cytotoxicity. Finally the cellular uptake of the NPs was seen. It was evident from the results that the multiple drugs loaded CuO NPs formed with PLGA coating were uniform, non-agglomerated in size ranging from 180 to 195 nm. The FTIR revealed no major changes in drug peaks. Encapsulation and loading efficiencies showed sufficient amount of drug being loaded into the NPs. The drug loaded NPs showed no change in size or zeta potential even after a period of 30 days. The cytotoxicity studies revealed significant reduction in toxicity after coating the surface treated with PLGA as evident from the microscopic analysis of cells. Hence the current study may be prioritized and further in vivo*/*in vitro studies may be carried out.

## Introduction

A subvariant of head and neck carcinoma, nasopharyngeal carcinoma is a rare kind of carcinoma which affects the population of Southeast Asia, China and USA in a major manner (Lam et al. 2016; Chua et al. 2016). It has been reported that nasopharyngeal carcinoma generally has three subtypes which affects adults and children separately (Sobin 1991). It has also been noted that conventional therapies to counteract nasopharyngeal carcinoma is accompanied by several detriments. The conventional therapy includes cytotoxic drugs treatment, radiation and finally surgical removal of the tumor (Sharma et al. 2016). Teratogenicity and cytotoxicity are two of the most severe detriments of anticancer chemotherapeutic agents (Sharma et al. 2016; Nobili et al. 2006). Furthermore, development of resistance against the drugs frequently results in increasing the dosage of the same, thus worsening the problem.

An alternate pathway to circumvent the toxicity and lessen the dosage of the anticancer drugs is by the means of encapsulating them in nanocarrier vehicle specifically targeting them to the targeted tissue (Sharma et al. 2016; Sun et al. 2008; Veiseh et al. 2010; Davis et al. 2010). The advent of nanotechnology provides an opportunity to synthesize nanocarriers in sub-micron range (1–100 nm) like liposomes, nanoparticles and cubosomes etc. which are extremely suitable for targeted drug delivery (Ahamed et al. 2015). Because of their sub-micron size they have enhanced penetrative properties which may disrupt the function of the targeted cells (Jiang et al. 2008; Magaye and Zhao 2012). These nanoparticles have increased time of deposition and retention in the body tissues (Madl and Pinkerton 2009). Metal nanoparticles especially copper oxide (CuO) nanoparticles are high on the radar of investigations because of their several interesting properties. CuO nanoparticles however have garnered interest owing to their unique properties of photovoltaics and photoconduction (Chang et al. 2005). However despite such interesting properties, the risk of humans coming in contact with CuO is undisputed. A significant challenge is estimating the toxicity of CuO nanoparticles and ways of diminishing it. There have been innumerable studies stating that leaching of Cu ions in the tissues may be a potential mechanism of increase in toxicity (Ahamed et al. 2015; Chusuei et al. 2013). Therefore in our study, a key challenge was to reduce the cytotoxicity of the CuO nanoparticles. We have therefore formed a coating of PLGA over the CuO nanoparticles to render them biocompatible. Alternatively natural substances have been used regularly to reduce the side effects of metal nanoparticles. Biocompatibility and non-immunogenic properties are associated with PLGA (Majumder et al. 2018).

Therefore, we reported the biosynthesis of novel CuO nanoparticle loaded with paclitaxel, docetaxel, coated with PLGA and conjugated with folic acid. It has also been studied that folate (folic acid) conjugated nanoparticles are utmost effective as cancer conjugation moieties (Samadian et al. 2016). Folate receptors have increased affinity towards folate moieties which are overly expressed in malignant cells when equated to regular cells (Khoshgard et al. 2014; Zhang et al. 2016). Doxorubicin and docetaxel have been used successfully used in chemotherapeutics of nasopharyngeal carcinoma and are established anti-cancer drugs. The main objective of our study was to reduce the cytotoxicity of the CuO nanoparticles by coating them with PLGA and hence synthesis of a biocompatible nanoparticle carrier which carried multiple drug to targeted tissue without causing any damage to surrounding tissues. In this study we established that PLGA coating over CuO nanoparticles seriously tweaked the surface properties of the nanoparticle and will improve the biodegradation of the nanoparticle inside body. Alongside the synthesis of nanoparticles, it has been suggested by scientific experts to characterize the particular nanoparticle before its toxicity studies (Chang et al. 2012). Hence characterization studies including micrographic analysis, in vitro drug release, encapsulation and loading efficiency, stability studies, zeta potential analysis, polydispersity index (PDI) of the PLGA coated drug loaded CuO nanoparticle was carried out too.

## Materials and methods

### Materials

PLGA (Resomer RG 85:15H), Polyvinyl alcohol (PVA, MW: 30000–70000), CuCl_2_ were procured from Sigma Aldrich, China/Docetaxel and doxorubicin were procured from Chengdu Mansite Pharmaceutical Co., Ltd. from Sichuan, China. Shanghai LeiDi biotechnology Co. Ltd. from Shanghai, China provided human nasopharynx carcinoma cells (HNE-1). RPMI medium 1640 procured from Gibco, Waltham, MA was used to culture the HNE-1 cells augmented with 10% FBS also procured from Gibco, Waltham, MA. The entire procedure was carried out at physiological temperature (37 °C) in an incubator with 5% CO_2_ & humidity for scheduled time period utilizing allied process.

### Preparation of PLGA coated folate mediated CuO nanoparticles (NPs)

Following some previously performed studies, to 50 mL of 1% water-soluble PLGA solution, a mixture of 0.25 mg of CuCl_2_ & 2 mL of sterile water was added to begin a reaction. Continuous stirring of the subsequent mixture was carried out for 6 h and the temperature at about 80 °C was throughout maintained which led to the development of PLGA-CuO NPs. The synthesis process was carried out using three varied concentrations of PLGA 0.5%, 1% and 2% to depict the advantage of PLGA coating over the CuO NPs during drug encapsulation and release respectively. The PLGA-CuO NPs synthesized with 0.5%, 1% and 2% PLGA concentrations were termed as C-1, C-2, C-3 respectively, and the NPs were obtained by the mechanism of freeze-drying. The procedure was adapted with finer modification from Kannan et al. (Varukattu et al. 2020) and (Vivek et al. 2014). Following this, 4.5 mg of folic acid was mixed in 5 mL of dimethyl sulphoxide (DMSO). Next, in a ratio of folic acid/DCC 1:1, continuous stirring was done for 2.5 h in a N_2_ atmosphere. Finally, PLGA-CuO NPs were added with continuous stirring in same conditions for another 2 h. Thereafter, modified FOL-PLGA-CuO NPs nanoparticles were finally twice washed with water followed by freeze-drying.

### Preparation of multiple drug loaded formulations

Anticancer drugs docetaxel and doxorubicin which are sparingly water soluble were loaded inside the FOL-PLGA-CuO NPs (C-1, C-2, C-3) respectively. The procedure was adapted from Kannan et al. (Varukattu et al. 2020) with definite modifications. 5 mg of freeze-dried C-1, C-2 and C-3 were added to 25 mL of Milli-Q water and by addition of 1 mL of doxorubicin and docetaxel mixture (concentration of drug was taken as 1 mg/mL each) within the resultant solution. Continuous stirring was carried out for 12 h at 37 °C. The FOL-PLGA-CuO NPs acquired with 0.5%, 1% and 2% concentration of PLGA were named as CD-1, CD-2 and CD-3 respectively. CD-1, CD-2, CD-3 were dialyzed for 24 h. against Milli-Q water The resultant suspension was investigated using UV Vis spectrophotometer and were freeze-dried. These free dried NOs were used to carry out additional experiments.

### Physical characterization of the PLGA-CuO NPs

The morphological analysis of the CuO NPs, FOL-PLGA-CuO NPs and CD-1, CD-2 and CD-3 were done both by scanning and transmission electron microscopy (SEM & TEM). Following dispersion in water, coating of the nanoparticles with gold was done on aluminium stub assisted with double sided carbon tape ensuing a drop method. Gold sputter coating unit was utilized for 10 s at 10 Pa vacuum to coat the respective nanoparticles. 30 kV was the distinctive acceleration potential used following which the image was shot at the selected magnification. EDX analysis was also done via SEM to check the presence of elemental copper.

TEM of the CuO NPs, FOL-PLGA-FA-CuO NPs and CD-1, CD-2 and CD-3 was done with high resolution Tecnai G2 30 (FEI, Netherlands) microscope reinforced with twin lens, power being 80 kV and the electron source being LaB6. Upon a copper grid (300 mesh) coated with carbon, a single drop of nanoparticle was positioned carefully with the excess being soaked up with an absorbent paper. 1% sodium phosphotungstate solution was utilized for staining the nanoparticles and photomicrographs were seen at magnification up to 1000,000 X. Air-drying and incubation of the NPs were carried out for 12 h following which the TEM images were perceived. The procedure was adapted with few changes from (Malamatari et al. 2015). FTIR analysis was carried out to determine the properties of the constructed NPs. The instrument utilized was IRAffinity-1S FTIR spectrophotometer (Shimadzu) with wavelength ranging from 400 to 4000 cm^−1^. This instrument provides 30000:1 ratio with an accumulation of single minute, neighbourhood of 2100 cm^−1^, and resolution of 0.5 cm^−1^ maximum. CuO nanoparticles, PLGA-CuO NPs and CD-1, CD-2 and CD-3 were reorganized in sterile water for purification before the procedure.

### Particle size, polydispersity index (PDI) and Zeta potential measurement studies

For calculation of mean diameter, size distribution (PDI) and zeta potential of CuO nanoparticles, FOL-PLGA-CuO NPs and CD-1, CD-2 and CD-3, Delsa Nano C Zetasizer was deployed. The readings were obtained at physiological temperature. Weighing and dispersion of the freeze-dried nanoparticles in Milli-Q water was done earlier for dilution along with achieving a suitable scattering intensity. Measurement of each sample was done thrice. Implementation of the experiment was carried out at 25 °C. The mean of three measured values of electrophoretic mobility was finally accepted as the optimum ζ potential values.

### Determination of encapsulation efficiency (EE%) and loading efficiency(LE%) of the nanoparticles

The procedure was followed with suitable changes from (Zhang et al. 2018; Wei et al. 2017) with changes to suit our experiments. CD-1, CD-2 and CD-3 were centrifuged at 10,000 rpm (High Speed Centrifuge, TGL-16B, Shanghai, China) for 20 min leading to the separation of docetaxel and doxorubicin from the NPs. With a 0.45-μm filter, the supernatant was collected.

An HPLC system (Agilent, US) having the following prerequisites was used for the investigation of free drugs in CD-1, CD-2 and CD-3: a TCC 300 C18 column (250 mm × 4.6 mm, 5 μm), a identifying wavelength of 425 nm and a mobile phase of 7.5:7.5:85, v/v of methanol/acetonitrile/0.4% aqueous phosphoric acid and the flow rate of 1 mL/min. After CD-1, CD-2 and CD-3 were dissolved in ethanol; the amount of the total drug loaded was calculated. The multiple drug encapsulation efficiency (EE) along with loading efficiency (LE) was the result of the following formulae:

1 $$ EE \left( \% \right) = Encapsulated drug \div Total drug \times 100\% $$

2 $$ LE \left( \% \right) = Total drug \div NPs \times 100\% $$

The equations were employed to decide the EE and LE% of CD-1, CD-2 and CD-3.

### Stability studies

The stability of CD-1, CD-2 and CD-3 were tested at room temperature where 10 mg of NPs were disseminated in 50 ml of Milli-Q water through sonication. Visual observation of settling of NPs were carefully made which was followed by size distribution measurement, polydispersity index and zeta potential of the NPs at steady time interlude of 10, 20, 30 days using the Delsa Nano C Zetasizer to note the aggregation of NPs.

### In vitro drug release from the NPs

Here in our study, we implemented a marginally divergent approach for the release of drugs in vitro into the medium. A dialysis bag (3 ml) was taken and 2.5 ml of each CD-1, CD-2 and CD-3 were loaded into it. For unimpeded dissolution outsized undissolved molecules were permitted to diffuse out through the dialysis bag. The dialysis bag was retained in 2.5 L of 10 mM phosphate buffer solutions (pH- 7.4 and 4.5) where after every 3 h, the buffer was renewed. The pH mimicked the cancer environment (4.5) & normal physiological pH (7.4) respectively. The buffer temperature was maintained throughout at 37 °C. At consistent time gaps of 4, 12, 24, 36, 48 and 72 h, 150 µl of solution was removed and renewed by same amount of fresh buffer. The drug concentrations in the NPs were estimated at 266 nm and were fitted to standard curve by means of UV–Vis spectroscopy. The absence of drug in the solution indicated a complete in vitro release of drug. In order to calculate the mean value, observations were made thrice.

### Statistical analysis

The data was recorded as mean with standard deviation (mean ± SD) and analyzed/calculated by Origin 8. Calculations were consummated with one-way analysis of variance (ANOVA). Statistically significant difference was pronounced with p value of < 0.05.

### In vitro cytotoxicity

#### Cell culture

Nasopharyngeal carcinoma CNE-2 cell line, the folate receptor negative cells were the chosen cell line for this study. CNE-2 cells were obtained from Chinese Cell Bank of the Chinese Academy of Sciences. In an atmosphere of humidified 5% CO_2_, the cells were retained in RPMI1640 medium (without folic acid) with 10% fetal bovine serum (FBS) at physiological temperature of 37 °C. While the cells were growing exponentially, they were incubated and seeded on a 96 well plate at 5 × 10^4^ cells/mL cell density.

### Cytotoxicity assay

After a cellular incubation period of 24 h, PBS (control), CuO NPs, FOL-PLGA-CuO NPs, CD-1, CD-2, CD-3, free doxorubicin and docetaxel were supplemented in the cell culture at a dose of 0.1 mg/ml. The dosage of 0.1 mg/ml was utilized for biological formulations. At 6 h time interval, the medium was withdrawn followed by PBS washing of the cells. Thereafter at room temperature, to each of the well, 20 μL of MTT solution (5 mg/mL) was being added after which 4 h incubation time was given. Once the incubation time was over, the absorbance was calculated at 492 nm by means of a microplate reader. The results were repeated for 5 times and articulated as mean ± SD.

### Live/dead cell assay for cytotoxicity

Cytotoxicity of the PBS (control), CuO NPs, FOL-PLGA-CuO NPs, CD-1, CD-2, CD-3, free doxorubicin and docetaxel were determined by live/dead cell assay. The Live/Dead cell assay kit was procured from Thermo Fisher Scientific, China. The method monitored was as per the directives mentioned by the company. The study was done with 5 mL of mixture of dyes (100 mg/mL acridine orange (AO) and 100 mg/mL Ethidium bromide, EtBr), supplemented in a 9 ml cell suspension having concentration of 10^5^ cells/mL on the coverslip for the microscope purposes. After incubation of 3–4 min, visualization of cells was done using a Nikon made fluorescence microscope with an excitation filter of 510–590 nm. The procedure was adapted from Kannan (Varukattu et al. 2020) and Vivek et al. (2014).

### DAPI staining

The CNE-2 cells were exposed to CuO NPs, FOL-PLGA-CuO NPs, CD-1, CD-2, CD-3, free doxorubicin and docetaxel as the protocol mentioned above. The NP and drug treated cells are fixed with methanol: acetic acid (3:1, v/v) before PBS wash. For staining the cells, 1 mg/ml DAPI was utilized and the cells were kept in dark for 30 min. A fluorescent microscope was utilized to study the images of the cells stained with DAPI using excitation and emission wavelength of 358 and 461 nm respectively.

### Impediametric studies

The device to measure impedance (ECIS) consisted of eight wells. Ten gold microelectrodes of 250 μm diameters were present in each cell which sensed the smooth flow of current through the solution. Each well had a volume of 600 μl with a substrate area of 0.8 cm^2^. Within a tissue culture incubator, the ECIS devices were incubated with RPMI 1640 media overnight. Confluent CNE-2 cell cultures were incubated from a cell culture mentioned above. Cells were incubated for 72 h and the following were added: PBS (control), CuO NPs, FOL-PLGA-CuO NPs, CD-1, CD-2 and CD-3 at the concentration of 0.1 ml. Incubation for 48 h was carried out and thereafter measurements of impedance values of the samples were carried out. The frequency range was designated from 100 Hz to 1 MHz in a logarithmic scale. Consequently, ZsimpWin (Ver. 3.10) software was used to fit the impedance data. Pradhan et al. (Pradhan et al. 2014) supplied the equivalent circuit for the impedance measurements explained in Fig. 5. RS & RI was the resistance of solution and charge transfer resistance respectively while CS and QM represented capacitance of water and interface impedance of cells.

### Statistical data analysis

The analysis of impedance values and investigations of cell assay were done in triplets to produce reproducible data and the data were represented with their equivalent relative standard deviations (RSD).

### Cellular uptake of the NPs

The procedure we adapted with modifications was of (Mo and Lim 2005) in our work. 5 × 10^4^ cells/mL of cell suspensions of HNE-1 and CNE-2 were seeded in 96 well plates. Thereafter when 80% cell confluency was attained, the cells were incubated with CD-1, CD-2, CD-3, free doxorubicin and docetaxel in RPMI 1640 for 6 h at 37 °C. Prior to incubation, docetaxel was coupled with fluorescent groups and we utilized NBD fluorophore for labelling as per the study of Dubois et al. (1995). At interval of 1 hour, for 3 times, the wells were rinsed with 0.05 mL of PBS with addition of 0.1 mL of culture medium. Afterwards, 0.05 mL of 0.5% triton in 0.2 N NaOH was applied as lysing agent for all wells. The intensity of fluorescence of free doxorubicin, CD-2 and CD-3 was observed and noted with a microplate reader at excitation and emission wavelength of 552 and 575 nm respectively. The intensity of fluorescence of free labelled docetaxel and CD-2 was noted at excitation spectra of 295 nm and emission spectra of 305 nm. The percentage of cellular uptake efficiency was deliberated according to the following formula:

3 $$ {\text{Cellular uptake efficiency}}\left( \% \right) = {\text{Fluorescent efficiency of samples}} \div {\text{Flourescent efficiency of control }} \times 100 $$

### Statistical data analysis

Complete statistics in the current study were articulated as means and standard deviation (mean ± SD) and analyzed by Origin 8 & Microsoft excel. Calculations were done using the one-way analysis of variance (ANOVA). The statistically significant difference was deliberated to be p value < 0.05.

## Results

### Physical characterization of the NPs

**Fig. 1:**
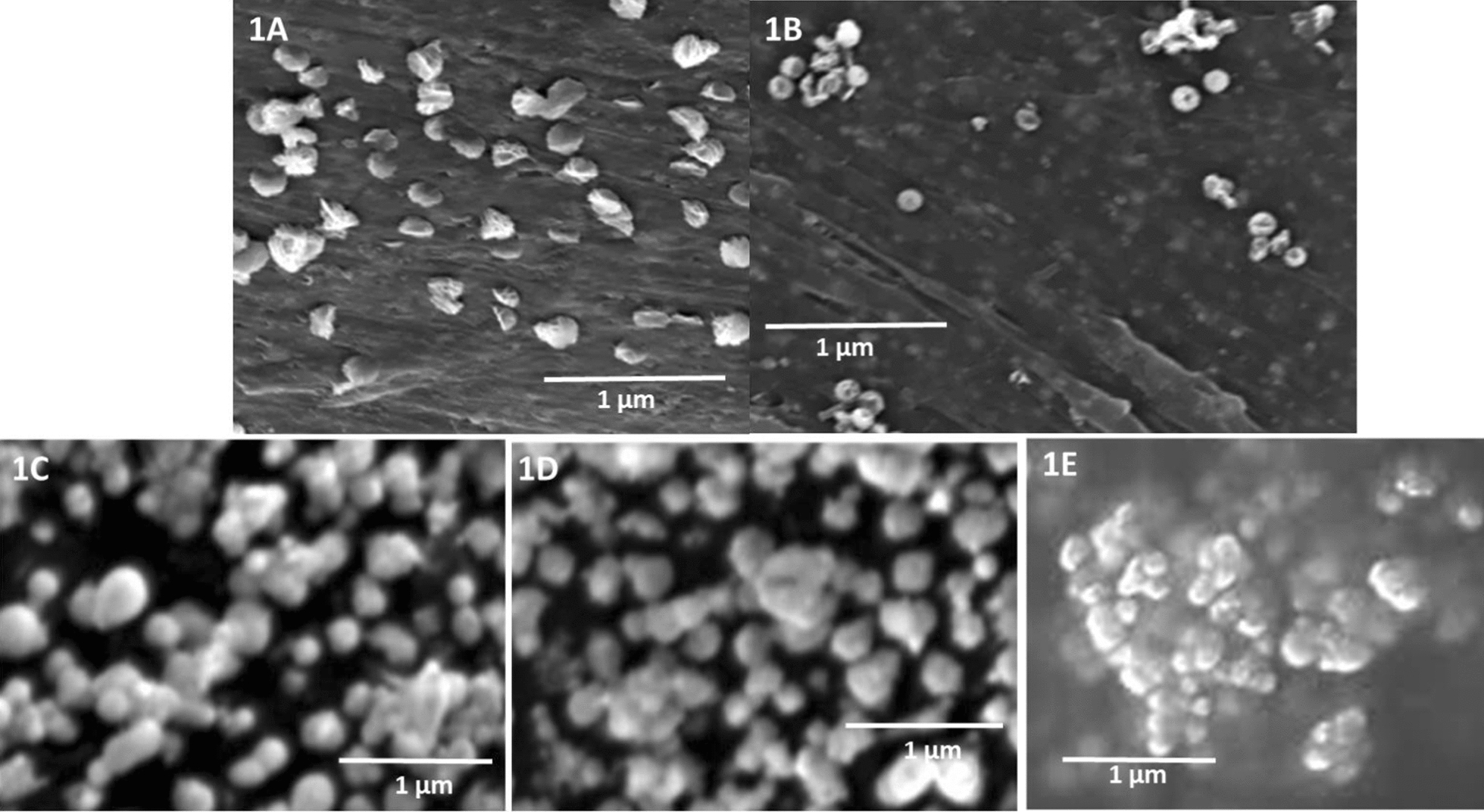
SEM of (**a**) CuO NPs; **b** FOL-PLGA-CuO NPs; **c** CD-1; **d** CD-2; **e** CD-3. Note the irregularities in size and shapes of CuO NPs in (**a**). Upon incorporation of drugs the shapes gradually become spherical with agglomeration of particles

**Fig. 2:**
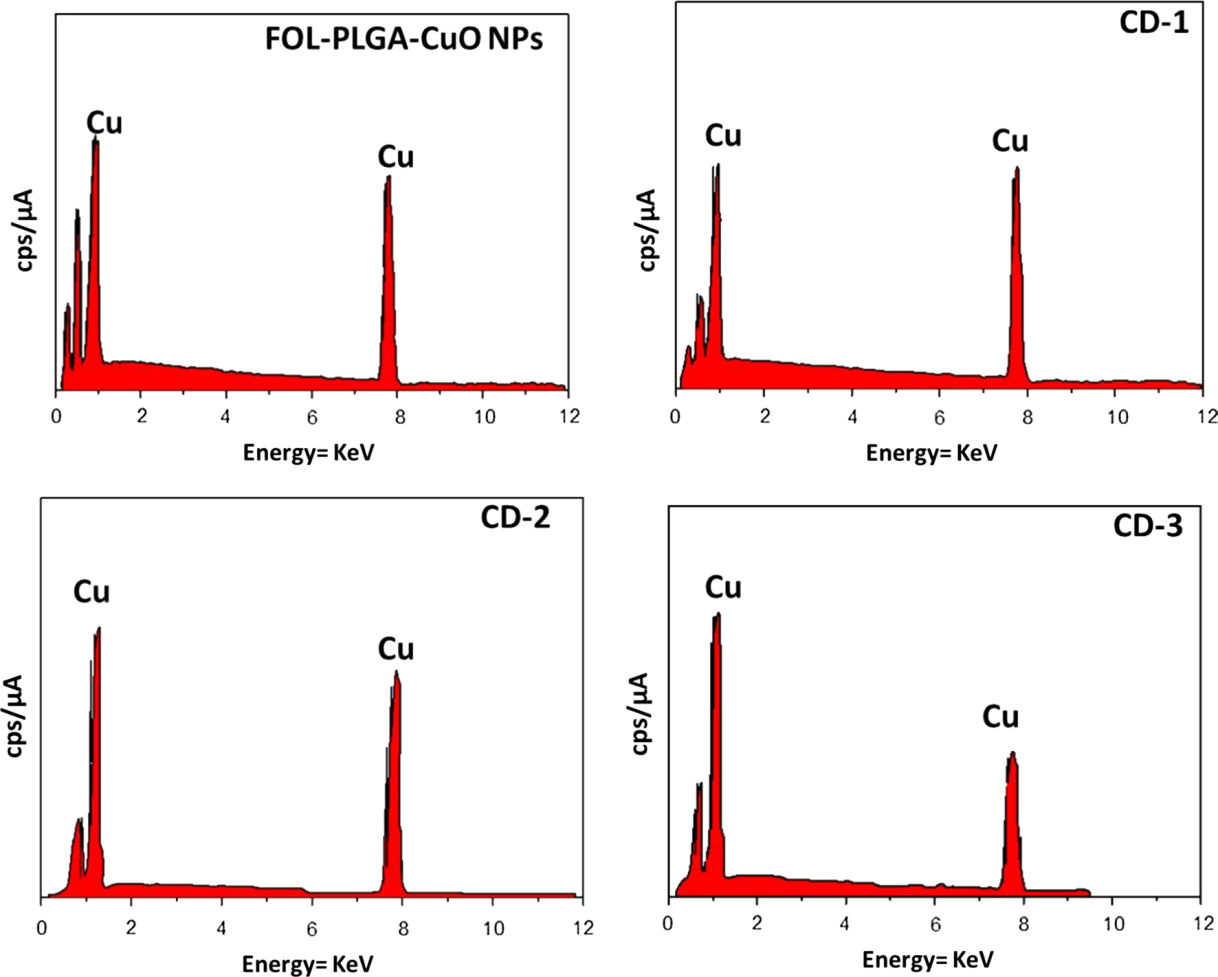
Presence of elemental Cu in CuO NPs, FOL-PLGA-CuO NPs, CD-1, CD-2 and CD-3. This indicates the incorporation of drugs in CuO nanoparticles

**Fig. 3:**
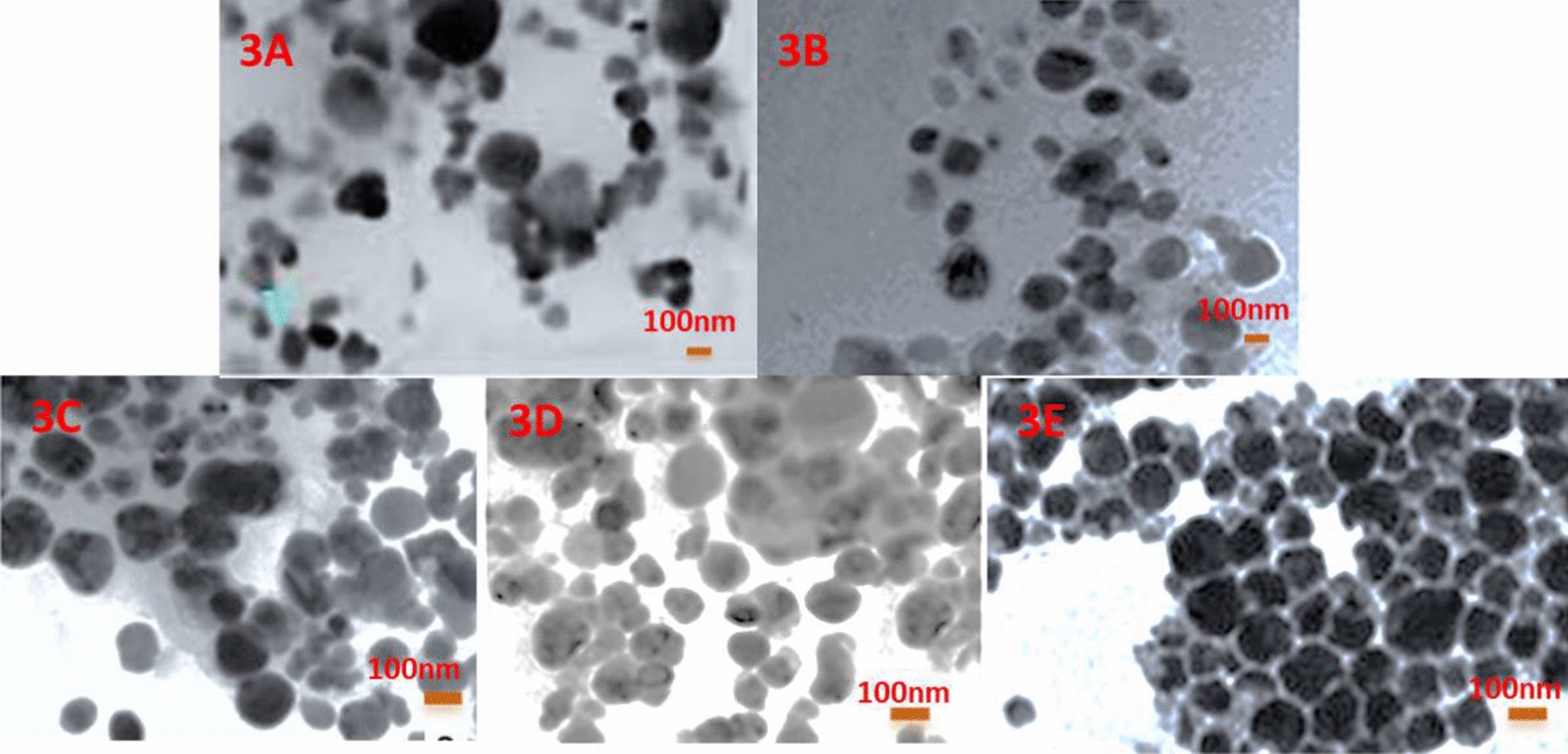
TEM of (**a**) CuO NPs; **b** FOL-PLGA-CuO NPs; **c** CD-1; **d** CD-2; **e** CD-3. Similar kind of irregularities as seen in SEM are also visualized in TEM micrographs of CuO NPs

The morphological analysis revealed the spherical structure of CuO NPs, FOL-PLGA-CuO NPs and CD-1, CD-2 and CD-3 via SEM. The synthesized NPs had uniform size distribution. The CuO NPS revealed irregularities in shape and size with slightly serrated edges as may be seen in Fig. 1a while following the coating of PLGA and folic acid over CuO nanoparticles revealed smooth NPs in Fig. 1b. Subsequently in Fig. 1c–e, smooth NPs were observed for CD-1, CD-2 and CD-3. There was no observed agglomeration or accumulation of the NPs. Elemental Cu was also detected via the EDX studies as seen in Fig. 2. Furthermore, the loading of the drugs docetaxel and doxorubicin were indicated by the TEM photomicrographs in Fig. 3. TEM photomicrographs reinforced the observation made BY SEM. The coating PLGA and conjugation with folic acid also influenced the size of the NPs. It was seen that while the CuO NPs were in the range of 115 nm, the size increased to 165 nm in case of FOL-PLGA-CuO NPs and in case of CD-1, CD-2 and D-3, the size ranged from 180 to 195 nm. The size, PDI and zeta potential values were indicated in Table 1. The zeta potential values of uncoated CuO NPs had positive charge (22.7 mV) which shifted to a negative charge on being coated with folic acid & PLGA (− 25.7 mV) and loaded with multiple drugs (− 26.8 mV).

**Table 1:** Size distribution, zeta potential and PDI of CuO NPs, FOL-PLGA-CuO NPs and CD-1, CD-2 and CD-3

Sl. no.		Size of nanoparticles (nm)	Zeta potential (mV)	Polydispersity index (PDI)
1.	CuO NPs	115.24 ± 2.3	22.7 ± 0.1	0.112 ± 0.01
2.	FOL-PLGA-CuO NPs	164.98 ± 1.3	− 25.7 ± 0.1	0.211 ± 0.03
3.	CD-1	180.23 ± 1.1	− 26.5 ± 1.3	0.345 ± 0.01
4.	CD-2	195.13 ± 1.6	− 26.3 ± 0.1	0.312 ± 0.02
5.	CD-3	189.45 ± 1.4	− 24.5 ± 0.2	0.223 ± 0.03

Note the shift in Zeta potential from +ve in CuO NPs to –ve in rest of formulations

**Fig. 4:**
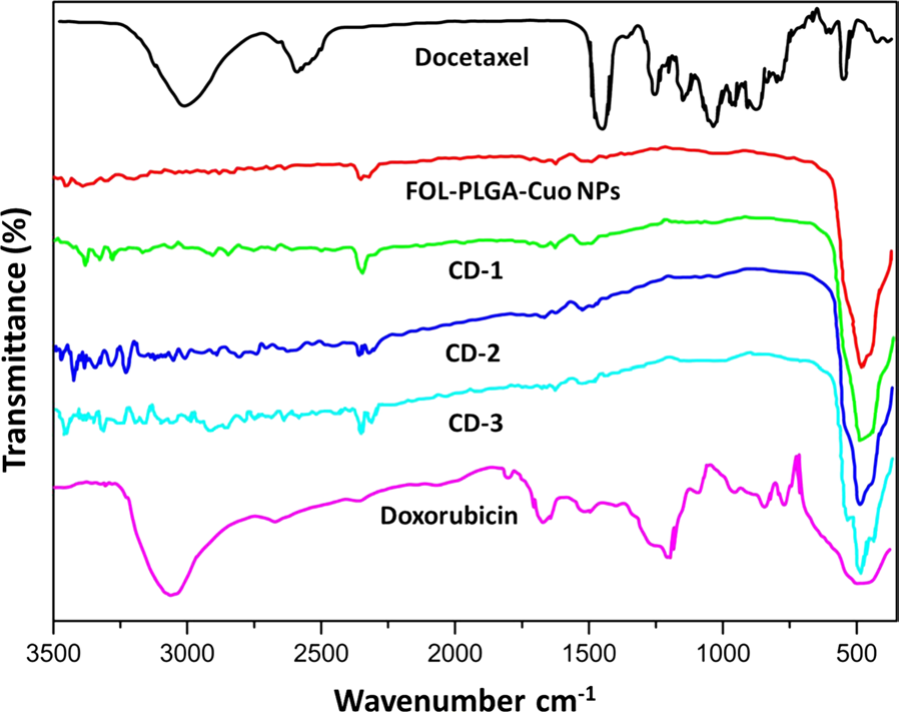
FTIR Spectra of free docetaxel, doxorubicin, CuO NPs, FOL-PLGA-CuO NPs, CD-1, CD-2 and CD-3. No new or drastic changes are seen in the spectra of CD-1, CD-2 or CD-3 which may indicate a change in function

FTIR data (Fig. 4) of free doxorubicin exhibited peaks at 3410 cm^−1^ due to N–H and O–H stretching vibrations at 3317 cm^−1^ due to primary amine structure. Similarly docetaxel revealed peaks at C=C asymmetric stretch of vibration and C=O variable weak intensity vibration at 721 cm^−1^ & strong intensity C=O stretching vibration at 1741 cm^−1^. The FOL-PLGA-CuO NPs showed folic acid conjugation with PLGA by a shift in peak at asymmetric stretching vibration of -NH_2_ in folate corresponds to the band at 1459 cm^−1^ and C=O stretching in carboxyl acids was seen at 1700 cm^−1^. CD-1, CD-2 and CD-3 showed shifted peaks below 3164 cm^−1^ caused by the drugs loaded onto NPs.

### Encapsulation and loading efficiency

The % of encapsulation and loading of drugs in CD-1, CD-2 and CD-3 were clearly denoted in Table 2. The loading of drugs by FOL-PLGA-CuO NPs was originally swift followed by a slow decline in the loading rate leading to complete inundation. The encapsulation efficiencies of CD-1, CD-2 and CD-3 were 70.15% ± 0.41, 82.15% ± 1.21 and 69.13% ± 2.12 respectively. There were noteworthy differences between the encapsulation of drug(s) in CD-1, CD-2 and CD-3 which may be related to the coating of PLGA over the nanoparticles. We will discuss this later. The drug loading efficiency CD-1, CD-2 and CD-3 were 39.5%, 42.45% and 34.14% respectively.

**Table 2:** Encapsulation efficiency (EE %) and loading efficiency (LE %) of CuO NPs, FOL-PLGA-CuO NPs and CD-1, CD-2 and CD-3 NPs

Sl. No.		EE%	LE%
1.	CD-1	70.15 ± 0.41	39.5 ± 2.23
2.	CD-2	82.15 ± 1.21	42.25 ± 1.34
3.	CD-3	69.25 ± 2.12	34.14 ± 2.31

There are noteworthy differences between the encapsulation of drug(s) in CD-1, CD-2 and CD-3 which may be related to the coating of PLGA over the nanoparticles

### Stability studies

The stability of the CD-1, CD-2 and CD-3 is depicted in Table 3. The size, zeta potential and PDI almost remain unchanged over a period of 30 days. No agglomeration of the NPs was observed during microscopy which was also confirmed by the stability studies. The slight changes in size may be attributed to rearrangement of the PLGA and folate on the surface of the NPS in neat and dense manner. However, a lot of factors like change in concentration of PLGA, centrifugation speed and time, pH may contribute for NPs stability.

**Table 3:** Size, zeta potential and PDI of CD-1, CD-2 & CD-3 after 10, 20 and 30 days during stability studies

	CD-1			CD-2			CD-3		
	Size (nm)	Zeta potential (mV)	PDI	Size (nm)	Zeta potential (mV)	PDI	Size (nm)	Zeta potential (mV)	PDI
10 days	181.23 ± 1.1	− 26.5 ± 1.3	0.345 ± 0.03	196.13 ± 1.6	− 26.3 ± 0.1	0.311 ± 0.02	190.13 ± 0.4	− 23.2 ± 0.2	0.221 ± 0.03
20 days	183.22 ± 1.3	− 24.3 ± 1.2	0.322 ± 0.04	197.33 ± 1.4	− 24.1 ± 0.3	0.310 ± 0.01	191.45 ± 1.3	− 22.6 ± 0.1	0.218 ± 0.01
30 days	184.3 ± 1.1	− 23.5 ± 0.3	0.311 ± 0.01	198.13 ± 0.2	− 22.3 ± 0.5	0.308 ± 0.03	192.15 ± 1.4	− 21.3 ± 0.3	0.217 ± 0.02

### In vitro release studies

**Fig. 5:**
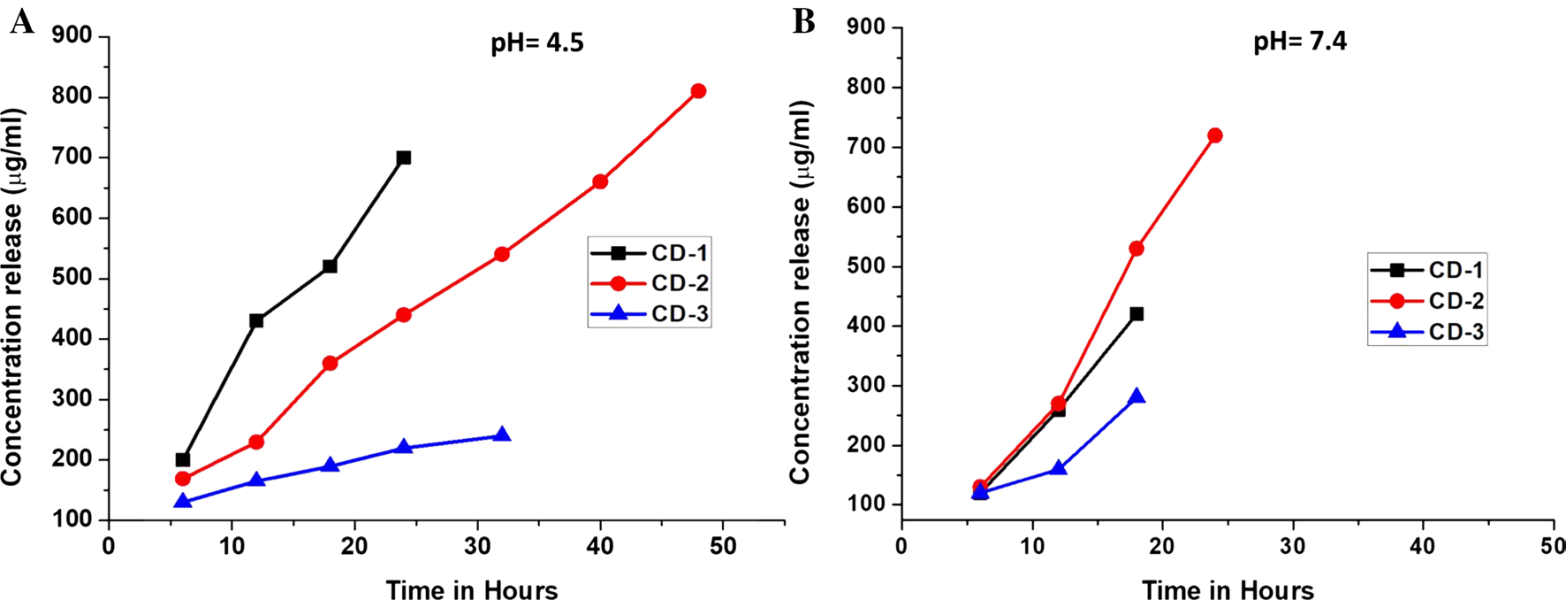
*in vitro* release of drugs: 5**a** In pH 4.5; 5**b** In pH 7.4. In the acidic pH mimicking tumor microenvironment, the ability to release higher amounts of drugs is seen for longer spell of time by CD-3

In vitro release studies were a big revelation for the percentage of PLGA to be utilized as coating over the surface of multiple drug loaded NPs as seen in Fig. 5a and b. It was observed in pH 4.5 that with CD-1 coating the release of the drugs from the NPs was fast and unhindered for 15 h. However it was observed that after 15 h the drug release from the NPs was slow and depleted within 25 h whereas in case of CD-2 coating of PLGA, the drug released continued in a slow and sustained manner up to 50 h. However, the important point was to note that in CD-3 coating of PLGA, the drug release was slow from the beginning and continued insignificant amounts most of the time which finally stopped altogether after 30 h. This was an extremely significant revelation. In pH 7.4, it was seen that for CD-1, the drug release was slow, in small spells for about 10 h only. For CD-2 it was slow and sustained for about 36 h and for CD-3, it was extremely less amount for 20 h only. It would be important to mention that pH 4.5 mimicked the tumor microenvironment whereas pH 7.4 mimicked normal physiological environment.

### Cytotoxicity studies

**Fig. 6:**
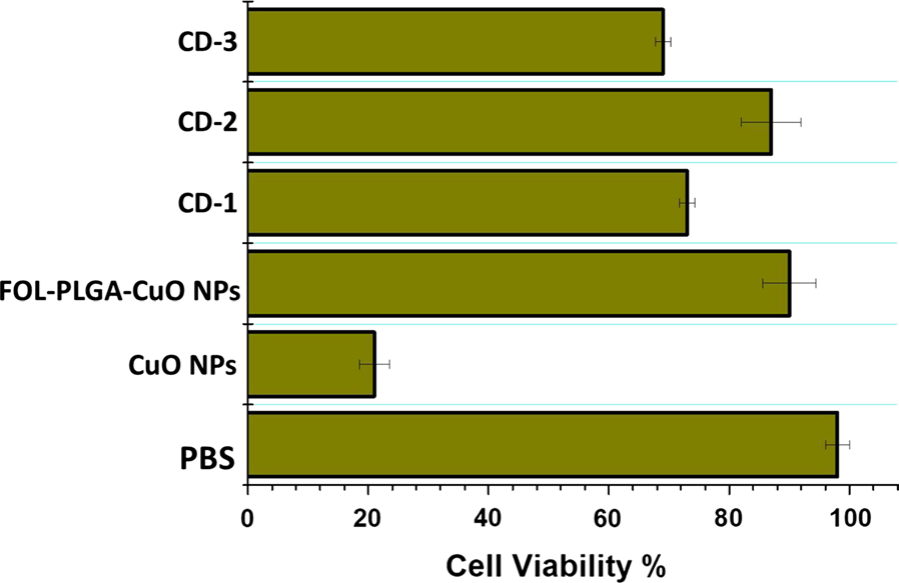
Cytotoxicity of PBS, CuO NPs, FOL-PLGA-CuO NPs, CD-1, CD-2 & CD-3. The cytotoxicity of CD-3 is quite reduced

As may be observed, a detailed cytotoxicity study of the PBS (control), prepared CuO NPs, FOL-PLGA-CuO NPs, CD-1, CD-2 and CD-3 NPs was executed utilizing MTT assay, AO/EtBr staining and DAPI staining of CNE-2 cells. It was seen that cells were cytotoxic in a % coating of PLGA dependent manner. The IC_50_ was obtained to be ~ 30 µg/ml for 48 h in CD-2, 27 µg/ml for 48 h in CD-3 and 22 µg/ml in CD-1. The CuO NPs were extremely cytotoxic and the viability of the cells decreased to 21% whereas in FOL-PLGA- CuO NPs the viability increased to 90%. On treatment with PBS, the cell viability was 97% as expected. In CD-1, CD-2 and CD-3, the cell viability was 72%, 83% and 69% respectively. The cells treated with PBS exhibited 90% viability. This was clearly exhibited in Fig. 6.

**Fig. 7:**
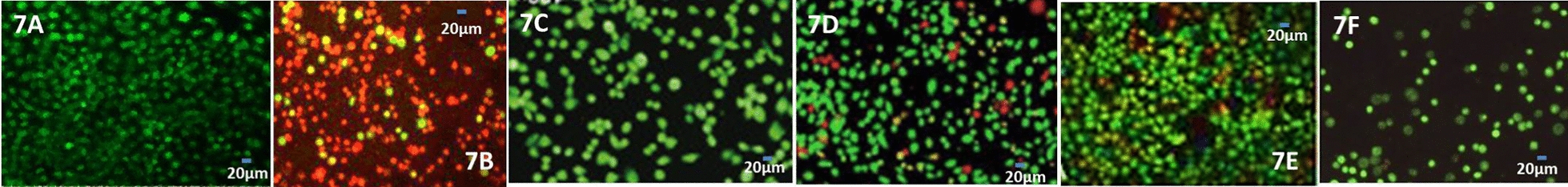
Live dead assay of CNE-2 cells on treatment with (**a**) PBS; **b** CuO NPs; **c** FOL-PLGA-CuO NPs; **d** CD-1; **e** CD-2; **f** CD-3. Note the reduced cell size and huge number of dead cells in (**b**) indicating potent cytotoxic effects much reduced in other formulations

The AO/EtBr reinforced the MTT assay results in Fig. 7. The photomicrographs revealed that the cells exposed to CuO NPs exhibited reduced cell size, dead cells, pcknosis, karyorrhexis which suggested the potent cytotoxic effect. There were also images of cell membrane blebbing and shrinkage of cells (Hengartner 2000). Uniform cells were observed in those treated with FOL-PLGA CuO NPs indicating much reduction of cytotoxicity of the NPs. There was decrease in the number of viable cells as indicated by green colour due to penetrability of AO in the samples treated with CD-1 which may be due to the uninhibited release of drug. The dead cells were indicated by red colour due to penetrability of both AO and EtBr. CD-2 & CD-3 exhibited maximum number of viable cells. The cells that were treated with CD-1, CD-2 & CD-3 did not depict any sort of damaged/destroyed cells or cell debris which may be an indication of reduction of cytotoxicity.

**Fig. 8:**
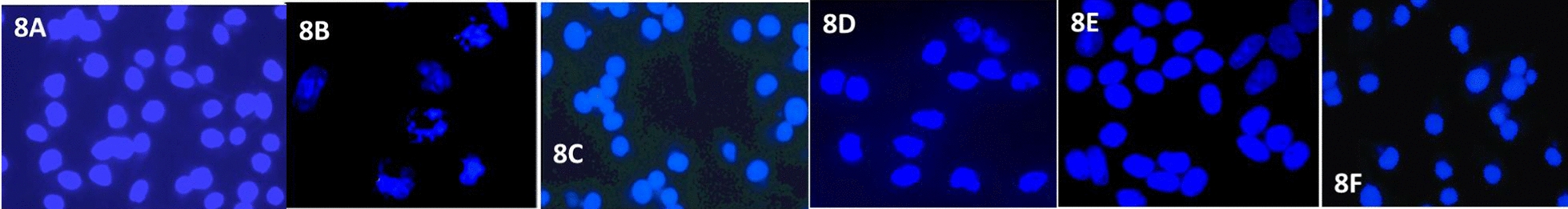
DAPI staining of CNE-2 cells on treatment with (**a**) PBS; **b** CuO NPs; **c** FOL-PLGA-CuO NPs; **d** CD-1; **e** CD-2; **f** CD-3. DAPI staining of cells treated with CuO nanoparticles show dead and disfigured cells not visible in other formulations

DAPI staining revealed a similar trend too in Fig. 8. DAPI is DNA binding nuclear cell stain which helped in identification of necrotic and live cells. Normal morphology of cells was perceived n treatment with PBS and FOL-PLGA-CuO NPs. The cells treated with CuO NPs revealed cellular suicide with prominent cell shrinkage, breaking and death. However upon treatment with CD-1, CD-2 and CD-3 showed limited cell deformations or death. Also, no loss of viability was evident.

### Impediametric studies

**Fig. 9:**
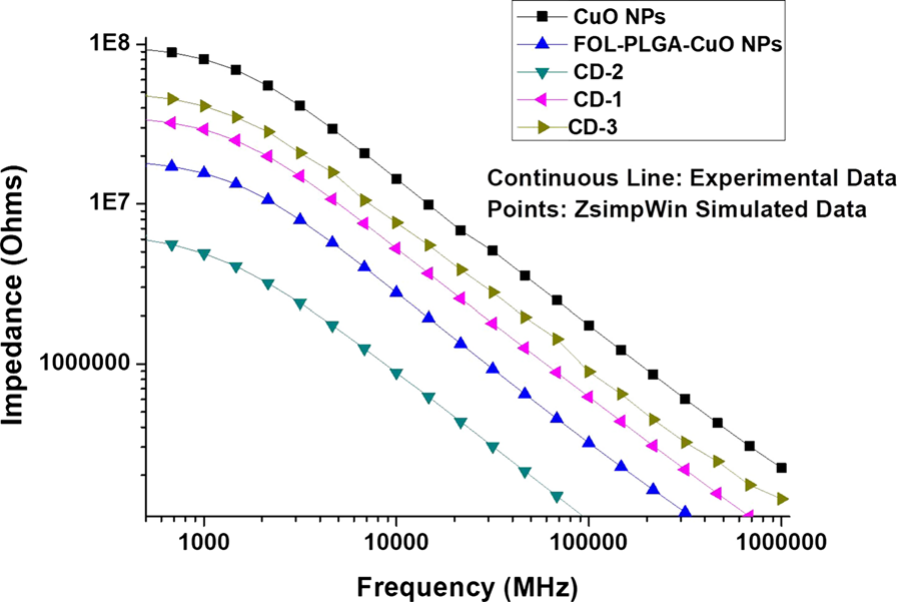
Impedance values of CNE-2 cells on treatment with CuO NPs, FOL-PLGA-CuO NPs, CD-1, CD-2 & CD-3

CNE-2 cells were chosen because of absence of folate receptor which may be equated to normal cells, plus they were easily cultured with proper documentation. Here we had purposefully chosen CNE-2 cells to view how the absence of folate receptors may affect the endocytosis-mediated delivery of NP formulations to targeted tissue. We theorized that progression of growth in cells of the CNE-2 will increase the cellular metabolism because of which there will be adequate cellular metabolism by-products which were extremely conductive in nature and may aid in decreasing impedance. We also assumed that the cytotoxic formulations may retard the growth of cells, thereby increasing impedance. It was clearly seen in Fig. 9 from the bode plot, that CuO NPs being the most cytotoxic there was increase in impedance and FOL-PLGA-CuO NPs along CD2 & CD-3 having least impedance values. CD-2 and CD-3 despite the drug release, offered least resistance to the growth of cells at lower frequency of 100 Hz. However, there is a decrease in the phase angle value up to 1 kHz and formation of plateau in the range of frequency from 1 to 10 kHz and then steady increase till 1 MHz. However at higher frequencies (4000 Hz), since current directly passes through the medium, the impedances dropped down. It was also proved that CuO NPs offer most resistance to the growth of CNE-2 cells owing to their cytotoxicity which was much reduced in PLGA-CuO NPs along CD2 & CD-3. It is an altogether different outlook in CD-1. Owing to a thin layer of PLGA coating and unhindered drug release, the impedance was low but still more than CuO NPs. However at higher frequencies, there was a dip in impedance values too.

### Cellular uptake studies

**Fig. 10:**
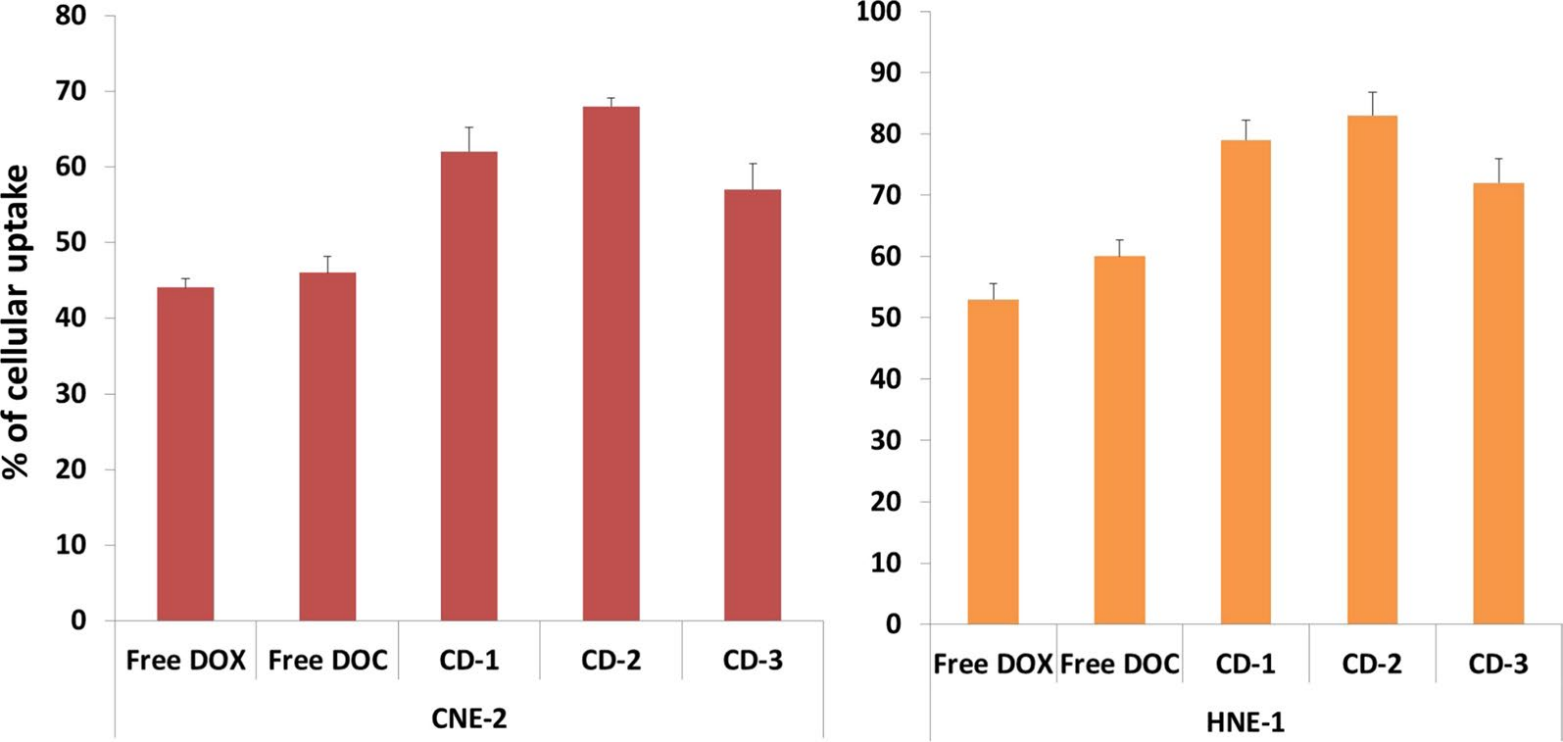
Cellular uptake of free docetaxel, doxorubicin, CD-1, CD-2 and CD-3. Reduced uptake of drugs and NPs by CNE-2 cells due to absence of folate receptors

Efficiency of cellular uptake of the NPs was an important dynamics for their synthesis. It was important to deliver the multiple drugs to the targeted tissue; hence two different nasopharyngeal cell carcinoma lines were designated for the study, folate positive (HNE-1) cells and folate receptor negative (CNE-2) cells. Figure 10 demonstrates the efficiency of cellular uptake of free doxorubicin, docetaxel, CD-1, CD-2 and CD-3. The free drug concentration was adjusted with the concentration of the drug in the NPs which was 1 mg/ml. Uptake of free doxorubicin and free labelled docetaxel by cells was consistently less than NPs in both cell lines. The HNE-1 uptake efficiency of free doxorubicin was 53%, docetaxel was 60% and CD-1 was 79%, CD-2 was 83% and CD-3 was 72% respectively. However when compared, the uptake of free doxorubicin, docetaxel and NPs in CNE-2 was significantly less than that of HNE-1. The CNE-2 uptake efficiency of free doxorubicin was 44%, docetaxel was 46% and CD-1 was 62%, CD-2 was 68% and CD-3 was 57% respectively. This may be accounted by the presence of folate receptors in the HNE-1 cells which triggers more cellular uptake of NPS that possess the folate ions.

## Discussion

A complete assessment of the physical and chemical characteristics of the synthesized NPs, that encompassed size, morphological analysis, structural & surface modifications along with coatings which resulted during the various synthesis techniques applied, needed to be analysed. Understanding this with the context of biology is essential to advance the pre-existing techniques which may need to undergo a complete transformation. There is an emerging need for development of new assays along with modification of the existing to suit appropriate control on quality assurance both at the theoretical research and contrived manufacturing production levels (Hussain et al. 2009). CuO NPs are usually known to be broad spectrum biocides which were known for their effectiveness against, bacteria, fungi and algae (Jia et al. 2012). But, despite being excellent microbicides, it had been reported widely that nanosized Cu generated greater cytotoxicity in cell lines and caused extensive damage (Sun et al. 2012).

Therefore, main challenge of our study involved minimizing the cytotoxicity of CuO NPs. The resolution of our study was to minimize CuO NPs cytotoxicity which otherwise are also excellent nanocarriers of drug to a particular targeted tissue. Hence after providing a coating of PLGA at different concentrations (0.5%, 1%, 1.5%), the characterization of NPs was carried out to predict the cytotoxicity albeit it was complex phenomena. The preparation of CuO nanoparticles in Milli-Q water rather than ethylene glycol or mixture of ethylene glycol/water may be conceptualized as one of the best methods of synthesis of the NPs (Kamalgharibi et al. 2016). It can be noted that the toxicological impact of nanomaterials varies from size and mode of fabrication, both in vitro and in vivo (Ranjan et al. 2020a; Ranjan et al. 2020b; Kumar et al. 2020; Dasgupta et al. 2019; Ranjan et al. 2019; Dasgupta et al. 2018; Ranjan et al. 2018; Aditi et al. 2018; Ranjan and Chidambaram 2016a; Dasgupta and Chidambaram 2016; Ranjan et al. 2016a; Dasgupta et al. 2016a; Ranjan et al. 2016b; Dasgupta et al. 2016b; Saxena et al. 2020).

In order to minimize the cytotoxicity of CuO NPs, the characterization of these may a significant role. For example, the size of the nanoparticles is an important consideration for toxicity studies. Numerous particle properties like surface reactivity and toxicity are derivatives of particle size of NPs. Particles with smaller size ensure a larger surface area-to-volume ratio when equated with bigger sized particles, and the particle numbers in a given mass is considerably increased. It has been assumed unanimously that NPs are known to possess more cytotoxicity than their larger counterparts. However, the relationship between physical characteristics of NPs and their toxicity goes way beyond that.

Any polymer-drug interaction is well represented by the FTIR data which in this case is descriptive in nature. After comparison of the free doxorubicin, docetaxel, CuO NPs, FOL-PLGA-CuO NPs, CD-1, CD-2 and CD-3 FTIR data, it was concluded that there was no changes in the structure of the drugs during encapsulation which may affect their efficiency as anticancer agents. There was no indication of loss of functional peaks or major shift in peaks. The negative zeta potential obtained in case of FOL-PLGA-CuO NPs, CD-1, CD-2 and CD-3 as against positive charge of CuO may led us to believe that negatively charged particles bind to the cationic sites of cells and may be the reason of higher cellular uptake.

Encapsulation and loading efficiency led us to conclude that the amount of PLGA used in the synthesis of NPs plays a significant part in the adsorption of docetaxel and doxorubicin. The coating probably prevented any leaching of drugs from the nanoparticles in case of CD-2 NPs. Where the coating in CD-1 was too thin to interrupt the uninhibited drug flow, in CD-3, the coating of 5% PLGA may be a hindrance to drug release for functionality as showed by the in vitro drug release experiments. pH also formed an important factor for the in vitro release of drugs. The ability to release higher amounts of drugs at pH mimicking tumor microenvironment was added as a favourable factor.

Cytotoxicity studies which were the prime goal of the current research were done in detail with different kind of staining procedures (AO/EtBr & DAPI). The cytotoxic effect of CuO nanoparticles were evidently seen in cell damage. However, the effect was much reduced after the three different concentration of PLGA coating was done. This may be indicative that the coating prevents the leaching of free copper ions in the cells which may cause the damage. The folate conjugation also improved the targeted drug delivery which is why the NPs were effectively taken up the HNE-1 cells. The cellular uptake studies also indicated that the folate conjugated with the receptors on the cellular surface and the intake of drugs may be carried out by the process of endocytosis.

The impediametric studies were also carried out to reinforce the cytotoxicity studies. The magnitude of impedance decreases progressively with frequency increase. This early disparity of slope at lower frequency range is because of the increased drug-cell interactions which leads to death of cells and hence their non-attachment from the surfaces of the electrode. The relative standard deviations (RSD) for different designs in experiment for cells treated with different drug concentration and untreated samples result below 10% which exhibited that the fabricated impedance biosensor devices are completely reproducible without errors. Hence it may, safely be concluded that the FOL-PLGA-CuO NPs, CD-1, CD-2 & CD-3 significantly deliver multiple drugs to the targeted cells without being acutely toxic to the normal/surrounding cells.

For designing an effective nanodrug carrier, one has to be categorically crucial to target cancer cells in addition to minimal toxicity toward the normal cells. CuO NPs as nanocarriers for multiple chemotherapeutic drug delivery to targeted tissue have garnered much consideration due to the theory that they escalate drug uptake by the cells via endocytosis by rather cancer cells than normal cells. However, cytotoxicity was a major concern for the use of CuO nanoparticles as nanovehicles for drug. In our study, we have loaded two potent anticancer drugs within CuO NPs in a two-step synthesis, and coated the NPs with PLGA followed by conjugation with folic acid to ensure greater targeting. A detailed characterization of the prepared nanoparticles was carried out to ensure reproducibility of the NPs or furthering the studies. The preparation of a cytocompatible CuO nanocarrier may encourage further in vitro and in vivo studies opening new pathways for transition of laboratory work to pre-clinical bench studies. Finally, it may be said that the study of this kind may be highlighted to minimalize the cytotoxic effect of CuO NPs on normal cells and consequently the health disorders that is tagged along. Our study sheds light on preparation of biocompatible CuO NPs for further pharmacological applications.
